# Instantaneous Relief in Cases of Blind Abscess

**Published:** 1907-12

**Authors:** James E. Keefe

**Affiliations:** Chicago, Ill.


### INSTANTANEOUS RELIEF IN CASES OF BLIND ABSCESS.

BY JAMES E. KEEFE, D. D. S., Chicago, Ill.

Instantaneous, and often permanent, relief from pain following infection from a pulpless tooth may be obtained by the use of equal parts absolute alcohol and water. Whether the pain is caused by an upper or lower tooth the treatment is equally effective.

The preparation can best be used with what is known as a watch case atomizer. Fill the atmoizer with the solution, then, having the patient recline the head a little, place the nozzle of the atmoizer into a nostril on the side where the trouble is, spray the solution back into the nostril twice and the pain will be relieved immediately. In some cases there will be a slight amount of gagging and the solution will come out through the mouth, but this comfort is only temporary.

If the pain should return within fifteen minutes a stronger solution, say 75 per cent. alcohol and 25 per cent. water, may be used and the treatment repeated, but usually the 50 per cent. solution will be sufficient and will permit you to open up the tooth and give the patient relief.

The watch case atomizer may be obtained from almost any large drug house. If you can not get one use any atomizer that will force a spray far back into the nose.—Items.

